# A virtual reality tool for the treatment of obesity: Study protocol of a randomized controlled trial

**DOI:** 10.1192/j.eurpsy.2021.1849

**Published:** 2021-08-13

**Authors:** P. Lusilla Palacios, D. Anastasiadou, J.A. Ramos-Quiroga

**Affiliations:** 1 Psychiatry, Hospital Universitari Vall d’Hebron. Universitat Autonoma de Barcelona, BARCELONA, Spain; 2 Psychiatry, VHIR, Barcelona, Spain; 3 Department Of Psychiatry, Hospital Universitari Vall d’Hebron. Universitat Autònoma de Barcelona, Barcelona, Spain

**Keywords:** obesity, virtual reality, motivational interviewing, cognitive behavioural therapy

## Abstract

**Introduction:**

Available evidence demonstrates that it is feasible to integrate Motivational Interviewing (MI) techniques with Enhanced Cognitive Behavioural Therapy (CBT) for the treatment of obesity and that this combined intervention has the potential to improve health-related outcomes of patients and to maintain behavioural changes over time. In addition, the use of Virtual Reality (VR) using embodiment techniques in the treatment of behavioural disorders has proved its preliminary effectiveness.

**Objectives:**

1) to adapt the embodiment tool for treating obesity in a clinical setting, and 2) to compare its preliminary effectiveness to usual care.

**Methods:**

A randomized control trial (SOCRATES project, funded by the European Union’s H2020 program under grant agreement No 951930) will be carried out with 66 participants with a Body Mass Index (BMI) >30, who will be split into two groups (control and intervention). The participants will be recruited from the external consultations of the Vall d’Hebron University Hospital. Readiness to change, BMI, dietetic habits and physical activity, self-perception of the body size, satisfaction with self-image and quality of life in relation to body image will be assessed before and after the intervention and at 4-week follow-up. Finally, variables related to the adoption of the VR tool in terms of perceived usability, user’s satisfaction and technology acceptance will be also evaluated.

**Results:**

Not yet available

**Conclusions:**

The study will provide an important advance in the treatment of obesity, first, by improving the effectiveness of available psychological treatments integrating embodiment, MI and CBT techniques, and second, reducing treatment duration and costs compared to conventional therapies.

**Disclosure:**

No significant relationships.

